# Towards Precision in Sarcopenia Assessment: The Challenges of Multimodal Data Analysis in the Era of AI

**DOI:** 10.3390/ijms26094428

**Published:** 2025-05-07

**Authors:** Valerio Caputo, Ivan Letteri, Silvano Junior Santini, Gaia Sinatti, Clara Balsano

**Affiliations:** 1 Department of Life, Health and Environmental Sciences, University of L’Aquila, P.le Salvatore Tommasi, 67100 L’Aquila, Italy; valerio.caputo@univaq.it (V.C.); ivan.letteri@univaq.it (I.L.); silvanojunior.santini@guest.univaq.it (S.J.S.); gaia.sinatti@graduate.univaq.it (G.S.); 2 Geriatric Unit, Department of Life, Health and Environmental Sciences, University of L’Aquila, P.le Salvatore Tommasi, 67100 L’Aquila, Italy; 3 Fondazione Francesco Balsano, via Giovanni Battista Martini, 00198 Rome, Italy

**Keywords:** sarcopenia, artificial intelligence, machine learning, multimodal analysis, diagnosis, molecular markers, circulating biomarkers, circulating proteome, nc-RNAs, metabolome

## Abstract

Sarcopenia, a condition characterised by the progressive decline in skeletal muscle mass and function, presents significant challenges in geriatric healthcare. Despite advances in its management, complex etiopathogenesis and the heterogeneity of diagnostic criteria underlie the limited precision of existing assessment methods. Therefore, efforts are needed to improve the knowledge and pave the way for more effective management and a more precise diagnosis. To this purpose, emerging technologies such as artificial intelligence (AI) can facilitate the identification of novel and accurate biomarkers by modelling complex data resulting from high-throughput technologies, fostering the setting up of a more precise approach. Based on such considerations, this review explores AI’s transformative potential, illustrating studies that integrate AI, especially machine learning and deep learning, with heterogeneous data such as clinical, anthropometric and molecular data. Overall, the present review will highlight the relevance of large-scale, standardised studies to validate biomarker signatures using AI-driven approaches.

## 1. Introduction

The rise in life expectancy is leading to an increase in elderly individuals in worldwide populations. By 2050, 1.5 billion individuals will be elderly, representing 16% of the world’s population (World Population Ageing 2023: https://www.un.org/development/desa/pd/sites/www.un.org.development.desa.pd/files/undesa_pd_2024_wpa2023-report.pdf (accessed on 23 January 2025)). Accordingly, sarcopenia affects 20% of the population aged 65–70 years and up to 40% of the over-80s, with a prevalence of 37–47% in the hospitalised elderly [1]. The ageing process is influenced by the alteration of several biological processes that contribute to the onset of sarcopenia, limiting independence and making older people frail. Age-related sarcopenia is defined as a progressive and generalised skeletal muscle disorder due to a decline in muscle mass and muscle function, strength and performance (including muscle fiber denervation and reinnervation) [2]. Predominantly affecting older adults, sarcopenia is frequently linked to chronic conditions such as cirrhosis, chronic renal failure, heart failure and chronic obstructive pulmonary disease (COPD), exacerbating disease prognosis and complications causing a worsening of the prognosis of complex chronic diseases and an increase in the number of complications that are often accompanied by cognitive decline [3,4,5,6]. Besides being a primary condition, sarcopenia can be strictly linked to nutritional status, thus contributing to the functional decline of the skeletal muscle system.

Interestingly enough, sarcopenia is underpinned by multifaceted pathophysiological processes, including mitochondrial dysfunction, systemic inflammation and hormonal dysregulation. These mechanisms contribute to chronic disease development, functional impairment and increased mortality, primarily through the progressive loss of lean body mass [7]. However, the related pathogenesis is still far from being completely understood, and many efforts are needed to improve this knowledge and pave the way for more effective treatments, management and a more precise diagnosis. On this topic, the assessment of sarcopenia has been addressed by different working groups worldwide that have produced three main guidelines (EWGSOP2 [8], AWGS 2019 [9] and SDOC [10]), which display some important differences in diagnostic cut-offs, that lead to discrepancies in assessing sarcopenia. Such a lack of alignment related to diagnostic criteria highlights the need to perform research on identifying novel, accurate biomarkers for avoiding the radiation risk of the current gold standard of sarcopenia, such as abdominal computed tomography (CT) scans.

Artificial intelligence (AI) can be helpful in finding disease signatures, taking advantage of integrating them with clinical data. On this subject, machine learning (ML), Artificial Neural Networks (ANNs) and deep learning (DL) are all topics that fall under the heading of AI and have gained popularity in recent years [11]. In particular, ML and DL are currently being applied in biomedical fields, especially diagnosis and risk assessment, the prediction of prognosis, follow-up and drug development, as well as having promising abilities for integrating data. For a technical and in-depth illustration of such AI frameworks, we refer the reader elsewhere ([12,13,14]). Briefly, ML-based models learn from data to make autonomous predictions or decisions, continually improving their accuracy over time. Within the ML domain, DL refers to a specialised subset that uses Artificial Neural Networks to model complex patterns and representations in large amounts of data [11].

This narrative review explores the transformative potential of AI, especially ML and DL, focusing on integrating multimodal clinical, anthropometric and molecular data, with particular attention to circulating biomarkers, in order to refine sarcopenia assessment and management.

## 2. Methodology for Selection of Studies

A literature search was conducted on PubMed and PMC (2010–2025) using combinations of keywords related to artificial intelligence and sarcopenia. Rather than following a formal systematic review protocol, studies were selected based on conceptual relevance and the significance of their findings. The illustrated studies employed artificial intelligence approaches for the management or integration of different kinds of data (clinical, anthropometric, instrumental and biological data) related to sarcopenia assessment and were selected based on their methodological approach and obtained results, which better allowed us to illustrate the state of the art.

It is worth noting that the non-systematic selection of papers, in literature review mode, may introduce publication and selection bias, as some studies have limited cohorts or no external validation. Moreover, many studies based on small samples may suffer from overfitting, so in this regard, we recommend the adoption of cross-validation and regularisation.

Section 3 includes studies found with the keywords “artificial intelligence” AND “sarcopenia” AND “data modeling”, “sarcopenia” AND “artificial intelligence” AND “data integration”, “artificial intelligence” AND “classification” AND “sarcopenia” “machine learning” AND “sarcopenia diagnosis”, “deep learning” AND “sarcopenia diagnosis”, “machine learning” AND “clinical data” AND “sarcopenia” and “deep learning” AND “clinical data” AND “sarcopenia”.

Section 4, instead, is built by employing the keywords “artificial intelligence” AND “molecular markers” AND “sarcopenia”, “artificial intelligence” AND “sarcopenia biomarkers”, “machine learning” and “transcriptome biomarkers” AND “sarcopenia” OR “muscle wasting”, “machine learning” AND “omic data” AND “sarcopenia” OR “muscle wasting”, “deep learning” AND “transcriptome biomarkers” AND “sarcopenia” OR “muscle wasting” and “deep learning” AND “omic data” AND “sarcopenia” OR “muscle wasting”.

Moreover, the present review aims at highlighting the potential of identifying novel disease biomarkers from liquid biopsy, exploiting the power of AI approaches. For this purpose, literature studies, investigating circulating markers of sarcopenia at protein, nucleic acid and metabolome levels, were selected to provide an overview of candidate biomarkers that, with the aid of AI, could be prioritised for further evaluation in the context of sarcopenia (see Section 5).

The literature search was thus performed on Pubmed and Pubmed Central, as well, using the keywords “circulating biomarkers” AND “sarcopenia”, “liquid biopsy” AND “omic data” AND “sarcopenia”, “circulating proteome” AND “sarcopenia”, “serum/plasma protein markers” AND “sarcopenia”, “circulating DNA” AND “sarcopenia”, “serum/plasma cell-free nucleic acids” AND “sarcopenia”, “circulating metabolome” AND “sarcopenia”, “serum/plasma metabolome” AND sarcopenia, “serum/plasma metabolites” AND “sarcopenia” and “circulating epigenetic signatures” AND “sarcopenia”.

## 3. AI for Comprehensive Data Integration in Sarcopenia

AI facilitates the integration of heterogeneous datasets—including electronic health records (EHRs), anthropometric profiles and biomarkers—going beyond conventional diagnostic approaches to uncover subtle interdependencies across data modalities. For instance, Luo et al. [15] achieved a high predictive accuracy by using machine learning (ML) models trained on EHR (i.e., data related to diagnostic assessment, drug administration and laboratory tests). The authors analysed data from 1304 adults who underwent musculoskeletal tests and classified patients according to the results. Data were extracted from the Indiana Network for Patient Care EHR 1 and used as a training source for five ML models, achieving an average area under the receiving operating characteristic curve (AUROC) above 90%. More recently, Onishi et al. [16] employed deep convolutional neural networks (CNNs) for building up an AI model, based on CT images and SMI, for sarcopenia diagnosis. A cohort of 3096 Japanese patients undergoing CT was split into training, test and validation cohorts. In the latter, the obtained accuracy was 0.88, showing good discriminatory abilities. The combination of parameters from multiple sources represents the aim of other studies. Specifically, in geriatric patients, a study by Wu et al. [17] was conducted to discriminate between the possible onset of sarcopenia in metabolic syndrome by using the arterial pulse spectrum (APS) and analyzing the data through ML analysis. In detail, radial blood pressure waveform (BPW) signals were measured in 133 subjects, dividing them into a control group and a group with possible sarcopenia. Two classification methodologies were used: ML analysis and a self-developed scoring system based on the 40 harmonic pulse indices of sarcopenia that are specifically associated. By applying threefold cross-validation, AUCs of 0.77 for ML and 0.83 for the scoring system were obtained, thus showing a promising discriminatory ability [17]. Furthermore, Castillo-Olea et al. [18] conducted a study on 166 older patients considering 99 variables, including geriatric assessment, demographic data and biochemical parameters. ML techniques with 10 classifiers were used to evaluate the predictors, and subsequently, the most performant model was determined. The results showed that age, Systolic Arterial Hypertension (SAH), the Mini Nutritional Assessment (MNA), the number of chronic diseases and sodium levels were the most important variables for predicting the onset of sarcopenia. The best classifier was the Radial Basis Function (RBF)–Support Vector Machine (SVM) [19], with an accuracy of 82.5%.

To overcome the limitations of existing sarcopenia screening tools, the integration of questionnaire data with biomarker variables was conducted by Wang et al. [20]. In such a study, the authors used data from the China Health and Retirement Longitudinal Study (CHARLS) [21]. The study employed robust statistical and ML techniques, including extreme gradient boosting (XGBoost), to achieve a good model with an AUROC of 0.759. Advanced feature selection and hyperparameter tuning are applied to enhance the predictive accuracy, reducing model complexity. Key predictors, including cognitive function (measured by the Mini-Mental State Examination, MMSE), drinking habits, education level and biomarkers (i.e., blood urea nitrogen), emerged, among others. Interestingly, the most relevant predictor for the classifier was represented by the decline in cognitive function, emphasizing the interplay between cognitive health and sarcopenia risk. The study by Zupo et al. [22] analysed an overall cohort of 1971 Italian participants aged ≥ 65 years using data derived from multiple anthropometric, clinical and biochemical variables related to sarcopenia. Dual-energy X-ray absorptiometry (DXA) was employed to evaluate variables such as Skeletal Muscle Index (SMI) and Appendicular Lean Mass (ALM) in combination with fluid biomarkers. Using three Random Forest (RF) [23] models, key predictors of sarcopenia were identified. RF models determined the most important variables related to sarcopenia: albumin, C-reactive protein (CRP), vitamin D and folates. Besides classifying overall sarcopenia, a study by Moroni et al. [24] applied logistic regression (LR) [12] analysis for subphenotyping the disease. In particular, the authors focused on a gender-imbalanced dataset of 1510 Italian patients (females, *n* = 1100; 72.85%) aged between 66 and 84 years to stratify sarcopenia-associated phenotypes such as sarcopenic obesity (SO), osteosarcopenia (OS) and osteosarcopenic obesity (OSO). Among the significant variables identified by LR for each condition, BMI represented a pivotal variable for sarcopenia, SO and OS. A broader use of ML models was conducted by Kang et al. [25] with a cross-sectional study employing classification algorithms, including LR, Support Vector Machine (SVM) [26], gradient boosting (GBM) and RF on a total of 4020 patients aged ≥ 65 years. The dataset used in this study comprises a population of 1698 (42.2%) male and 2322 (57.8%) female patients. It is combined with the Korea National Health and Nutrition Examination Surveys (KNHANES) [13] data, collected from 2008 to 2011. The selection of variables was carried out using a feature selection process that considered anthropometric features, such as BMI and waist circumference, nutrient intake (vitamin D, niacin) and haematological parameters. It is worth noting that the RF model accurately identified the same 17 factors selected by a panel of experts as significant (five orthopaedic surgeons, two nutritionists and one physician).

Finally, the combined integration of different AI methodologies, such as “traditional” machine learning algorithms, such as RF, SVM and XGBoost, offers partial interpretability through feature-importance metrics and performs robustly on moderate-sized datasets with relatively low computational demands. However, they depend heavily on manual feature engineering and may fail to capture highly non-linear relationships or process unstructured data without extensive pre-processing.

On the other hand, deep learning (DL) models provide end-to-end learning that automatically extracts complex representations from raw inputs (e.g., physiological signals) and scale effectively with large, heterogeneous datasets, yet they are inherently “black boxes” requiring additional explainability tools (e.g., SHAP [27], LIME [28]), they demand substantial computational resources (e.g., GPUs) and large cohorts for training, and they are prone to memorising training data in the absence of sufficiently large cohorts carrying a high risk of overfitting or in the absence of rigorous regularisation.

Nevertheless, these approaches appear to represent a novel opportunity for improving the identification of sarcopenia. For instance, an innovative study by Zhang et al. [29] combined linear and deep learning components, using Wide and Deep (W&D) [30] on data extracted from West China Health and Aging Trend (WCHAT) [31] cohorts, used for model training and testing, whereas the Xiamen Aging Trend (XMAT) cohort was employed for external validation. The study’s methodology highlighted the reduction of 109 features to 12 key predictors (mid-upper arm circumference (MAMC), calf circumference (CC), triceps skinfold thickness (TSF) and the AST/ALT ratio). The W&D model achieved exceptional diagnostic accuracy (AUC = 0.970, ACC = 0.911) in external validation, outperforming the other models and demonstrating its robustness and utility, further supporting that a hybrid design can be particularly effective for capturing the nuances of the data. Aiming to identify cancer patients at high risk of sarcopenia, a study developed an unsupervised approach that integrated data from the NRS-2002 and SARC-F questionnaires, anthropometric measurements and body composition parameters assessed by BIA. To this end, the authors analysed a cohort of 879 patients previously characterised within the NUTRISCREEN project. Using principal component analysis (PCA) followed by k-means clustering, three distinct patient clusters were identified, each with varying profiles of muscle mass, cellular integrity and fluid distribution. Subsequent multivariable analysis revealed that advanced age, certain cancer types (lung, gynecological and gastrointestinal cancers) and the occurrence of comorbidities (particularly diabetes) and malnutrition were significantly associated with an increased risk of sarcopenia. Notably, this study also found a higher prevalence of women in the high-risk cluster identified by PCA, emphasizing the need to consider sex as a contributing factor in sarcopenia risk assessment [32]. In line with the objective of the previously mentioned study, Gu et al. aimed at employing AI to predict the risk of sarcopenia in a cohort of 231 post-surgical gastrointestinal cancer patients, 155 of whom eventually developed sarcopenia. In this case, both an XGBoost model and LASSO regression with cross-validation were applied. The XGBoost model yielded the best performance, achieving an AUROC of 0.98, and identified serum albumin, diabetes as a comorbidity, type of surgery, nutritional score and Eastern Cooperative Oncology Group (ECOG) performance status as the relevant predictive variables. Notably, SHAP analysis was used to enhance model interpretability, highlighting serum albumin, nutritional status and diabetes as the key factors in predicting sarcopenia risk, thus aligning overall with the obtained outcomes by Porciello et al. [32]. These findings underscore the importance of comprehensive patient profiling in improving sarcopenia assessment.

The main applications of AI for the diagnosis of sarcopenia are summarised in Table 1, which reports the characteristics of the datasets, the artificial intelligence models used and the performance metrics obtained.

## 4. Application of AI for Identifying Molecular Biomarkers

The absence of consensus among the guidelines concerning the assessment highlights that improving the knowledge of etiopathogenesis to find novel, accurate biomarkers is crucial. Thus, the exploitation of AI approaches to facilitating the identification of specific biomarkers for sarcopenia is a promising avenue. Indeed, some efforts have been made to pursue this purpose. Chen et al. [34], in their study, combined public transcriptomic data from the Gene Expression Omnibus (GEO) (GEO: https://www.ncbi.nlm.nih.gov/geo/ (accessed on 23 January 2025)) datasets related to muscle tissue (GSE1428 and GSE136344) to identify differentially expressed genes (DEGs) between young and elderly individuals. Using LASSO logistic regression and SVM-RFE algorithms, the study selected eight candidate genes, namely, *TPPP3*, *C1QA*, *LGR5*, *MYH8* and *CDKN1A* that were found among the upregulated genes and *SLC38A1*, *SERPINA5* and *HOXB2* that were found downregulated. Bioinformatic predictive analyses, including Gene Set Enrichment Analysis (GSEA) and protein–protein interaction (PPI) showed that the candidate genes were closely interconnected and associated with critical biological processes in muscle tissue homeostasis, such as muscle myosin complex, retinoic acid binding and hormone receptor activity. The diagnostic accuracy was evaluated by ROC curve analysis, with all eight genes achieving an area under the curve (AUC) greater than 0.7. Similarly, the main goal of the study by Lin et al. [35] was to identify potential genetic biomarkers to develop a diagnostic model for sarcopenia. For this purpose, the study focused on four GEO microarray-based datasets, consisting of transcriptome data from muscles. In particular, three of these datasets, muscle biopsy-based datasets (GSE8479, GSE9103 and GSE38718), were combined to create a training dataset, while the GSE1428 dataset was used as a validation dataset (see Table 2). Overall, the authors identified 107 sarcopenia-related DEGS, which PPI analysis and Kyoto Encyclopedia of Genes and Genomes (KEGG) interrogation allowed them to be found as primarily enriched in the FoxO and AMPK signalling pathways. Using an RF classifier, authors prioritised seven key genes to be evaluated for their diagnostic potential (*MT1X*, *FAM171A1*, *ZNF415*, *ARHGAP36*, *CISD1*, *ETNPPL* and *WISP2*). An Artificial Neural Network (ANN) was used to build a diagnostic model, which obtained an AUC of 0.99 and 0.85 in the training and testing datasets, respectively, showing that three of these genes were mainly important for the model (*CISD1*, *ETNPPL* and *WISP2*). The work of Chung et al. [14], based on three transcriptome datasets (comprised within the GSE111017) in which profiles of 17,339 genes were obtained from 118 subjects (of whom 32 had sarcopenia), applied ML models, including RF, extreme gradient boosting (XGBoost) [36], Adaptive Boosting [37] and Deep Neural Networks (DNNs) to identify 27 relevant genes. Among these, a four-layer DNN (DSnet-v1) was the most performant model for the diagnosis of sarcopenia (Table 2). Interestingly, this study included subjects of different populations (i.e., European, African and Asian ancestry) and allowed the authors to investigate common and population-specific expression profiles of sarcopenia, for instance, *CENPC* was found upregulated only in European and Asian samples. Based on the above-illustrated works, the employment of AI approaches for dissecting relevant genomic signatures of disease within the transcriptome is proving to be a fertile area of research to identify candidate biomarkers. Besides profiling the transcriptome, it is important to note that the dysregulation of gene expression in sarcopenia may also be due to the alteration of epigenetic elements, which may represent themselves as powerful signatures connected to the disease. On this subject, Ahn et al. [38] investigated DNA methylome profiles of sarcopenia in middle-aged Korean men. The authors developed an ML model with high diagnostic accuracy using data from 509 male participants of the Korean Genome Epidemiology Study Health Examinee (KoGES) cohort. Participants were classified as sarcopenic based on appendicular skeletal mass index (ASMI) and handgrip strength (HGS). ML techniques were employed. In particular, a model based on recursive feature elimination with cross-validation (RFECV) was able to identify more than 200 differentially methylated CpG sites, and, among them, 8 were selected as able to accurately discriminate sarcopenic patients (AU-ROC = 0.94), according to the ensemble model with majority voting that was used for evaluation. Interestingly, such methylation profiles were found to be positively associated with age and negatively correlated with ASMI and HGS. For a more concise observation, we listed the molecular biomarkers identified by AI techniques in Table 2, with a focus on key findings and their validation analysis.

## 5. Circulating Biomarkers: Update and New Frontiers for AI in Sarcopenia Research

It is worth noting that liquid biopsy can provide a comprehensive picture of sarcopenia features in patients, shedding a glimmer of light on altered systemic mechanisms, such as immunity and age-related hormonal changes [39]. In addition, the damaged muscle can modulate the secretion of various inflammatory factors, such as cytokines and myokines (such as IL-6 or TNF-α) that exert paracrine and endocrine effects [40,41]. Bearing this in mind, several studies have tried to assess circulating biomarkers, including cf-DNA, nc-RNAs (especially microRNAs), proteins and metabolites to discriminate sarcopenic subjects from non-sarcopenic ones. We summarize in Table 3 the most significant results from studies aiming at associating circulating molecular signatures with sarcopenia. Unfortunately, such studies were heterogeneous in terms of methodology, including targeted and omic approaches, sample size and clinical and demographic features, not only leading to controversial or inconclusive results but also impairing the use of AI in the analysis of the obtained data. Moreover, various clinical studies on circulating biomolecules enrolled only male or female patients, even if it is known that results may be influenced by biological sex [42,43,44,45,46].

As a matter of fact, hormone profiles, the influence of the X-chromosome and sex-specific gene regulation can influence sarcopenia. Importantly, all the existing guidelines take into consideration sex differences, suggesting different cut-offs for strength and physical performance tests between males and females. The heterogeneity underlying different studies is further highlighted by two works that aimed at performing systematic reviews to assess the power of circulating miRNAs’ profiles related to sarcopenia. In particular, both works agree on the limitation to perform a robust meta-analysis integrating heterogeneous studies focusing on such nc-RNAs [47,48,49,50]. To date, the investigation on sarcopenia-associated circulating protein profiles has shown high heterogeneity and controversial results, too. This consideration is consistent with the outcomes obtained by a meta-analysis on four studies (of which two focused on circulating proteins) showing how protein profiles were not similarly modulated [51].

Nevertheless, exploring nc-RNAs as well as protein profiles can still be a useful approach to unveil insights into extra muscle signatures of sarcopenia. Again, even though metabolomics represents a powerful tool for assessing dynamic alterations related to disease [52], such studies have reported inconsistent data to date. For instance, although lipids represent the most differentially abundant metabolites, different profiles were detected depending on the study. Remarkably, among the studies summarised in Table 3, the differentially modulated signatures, which were evaluated for diagnostic power, raised at best an AU-ROC of 0.819 [42,45,53,54,55,56]. Such data support the necessity of integrating big data derived from biological and clinical studies. On this subject, AI could represent the right tool to achieve this goal. Bearing this in mind, as a first step towards the inclusion of circulating signatures in such approaches, we make the effort to summarize in Table 3 the most significant or accurate findings of each considered study.

**Table 3:** A summary of circulating signatures found as associated with sarcopenia. The specific signature, the biological fluid of origin and the detected profile are reported together with the related study reference.

Circulating Signature	Biological Source	Expression Profile	Ref.
D3-creatinine	urine	downregulation	[57]
ALDOA	serum	upregulation	[58]
CTSD	serum	upregulation	[58]
P3NP	serum	upregulation	[59]
IL6	serum	upregulation	[60,61]
TNF	serum	upregulation	[61]
CAF	serum	upregulation	[62]
VCAM1	serum	upregulation	[63]
GDF15	serum	upregulation	[64]
CETP	serum	upregulation	[65]
APOA2	serum	downregulation	[65]
IGF1	serum	downregulation	[66]
GH	serum	downregulation	[66]
Cf-mtDNA	plasma	high levels	[53]
miR-28-5p	plasma	upregulation	[45]
miR-1-3p	plasma	upregulation	[54,67]
miR-133a	plasma	downregulation	[44]
miR-133a-3p	serum	downregulation	[68]
miR-200a-3p	serum	downregulation	[68]
miR-434-3p	plasma	downregulation	[44]
miR-455-3p	plasma	downregulation	[44]
miR-486	plasma	downregulation	[55]
miR-146a	plasma	downregulation	[55]
miR-21	serum	upregulation	[69]
traumatic acid	plasma	high levels	[70]
ceramides	plasma	high levels	[71]
sphyngomielins	plasma	high levels	[71]
sphyngomielins	plasma	high and low levels depending on lipid	[72]
sterol ST(d14:0/25:5)	plasma	high levels	[72]
phosphatidylcholines	plasma	high and low levels depending on lipid	[72]
phosphatidylserines	plasma	high and low levels depending on lipid	[72]
PI 32:1	plasma	high levels	[73]
isoleucine	plasma	low levels	[73]
1-methylhistamine/3-methylhistamine	plasma	high levels	[73]
carnosine	plasma	low levels	[73]
creatinine	plasma	low levels	[73]
arginine	serum	low levels	[43]
cystin	serum	low levels	[43]
taurin	serum	high levels	[43]
hypoxanthine	plasma	high levels	[56]
hypoxanthine	serum	high levels	[74]
L-2-amino-3-oxobutanoic acid	plasma	low levels	[56,74]
PC(14:0/20:2(11Z,14Z))	plasma	low levels	[56]
LysoPC(17:0)	plasma	low levels	[56]
palmitic acid	plasma	low levels	[56]
mannose	serum	high levels	[74]
galactose	serum	high levels	[74]
triethanolamine	serum	low levels	[74]
homogentisic acid	serum	low levels	[74]
oleoyl ethanolamide	plasma	high levels	[42]
stearoyl ethanolamide	plasma	low levels	[42]
docosahexaenoylethanolamide	plasma	low levels	[42]

## 6. Discussion

Improving the assessment of sarcopenia remains a critical challenge due to the lack of harmonised diagnostic criteria across major guidelines (EWGSOP2, AWGS 2019, SDOC). While consensus exists on evaluating muscle mass, strength and physical performance [75,76], the variability in threshold definitions hampers the comparability and reproducibility of clinical and research findings. This discordance not only impairs patient stratification but also the development of universally applicable diagnostic tools. Besides fostering biomedical research on accurate and easy-to-access novel biomarkers, AI represents a novel and promising approach to overcoming these challenges by integrating heterogeneous datasets and revealing patterns within multimodal data.

As a matter of fact, the multifaceted nature of sarcopenia claims for a 360-degree picture of patients, thus encompassing data from clinical evaluation, such as proximate and remote pathological and pharmacological history as well as vital clinical parameters (e.g., heart rate, blood pressure), for taking into consideration the overall health status and occurrence of comorbidities, especially in elderly patients; anthropometric measures, such as BMI, waist–hip circumference, height and weight, mid-arm area muscle circumference (MAMC) and body mass composition; muscle mass evaluations and the degree of myosteatosis by computed tomography or ultrasonography, respectively; muscle strength and physical performance evaluations that specific tests can obtain and nutritional status assessment. Biomolecular data, especially those resulting from liquid biopsy, as mentioned above, can be exploited to improve knowledge on sarcopenia pathogenesis as well as ameliorate precision while limiting the invasiveness of the assessment. Therefore, including all these multimodal data into advanced diagnostic tools will allow for a comprehensive evaluation of patients in the hospital setting, thus enabling the identification of at-risk patients together with sarcopenia aetiology (age-related sarcopenia or secondary sarcopenia due to chronic illnesses or malnutrition) and patients’ stratification in terms of progression and severity. Such evaluation will pave the way for more effective and safe treatment of sarcopenia in light of the patient’s own features.

However, crucial limitations exist that hamper the clinical application of AI for sarcopenia diagnosis and management. As mentioned in the present review, one important limitation to achieve such a goal is the heterogeneity underlying the data (tabular clinical data, images, questionnaire results and molecular data) and the need to standardise them to facilitate the management. Furthermore, there is still a need to optimise and design specific AI models for each different field of application, especially with regard to biomedical issues. In fact, AI-based frameworks are applied indiscriminately for the analysis and integration of heterogeneous or limited datasets, so it is essential to evaluate and critically contextualise the real impact of these technologies. Consistently, several studies that used ML/DL models based on relatively small and homogeneous cohorts limited their generalisability to specific populations, ranges of ages, etiologies and even sex [15,32].

Moreover, many studies are conducted on small or homogeneous cohorts, thereby restricting the generalisability of the models: Zupo et al. [22] analysed exclusively Italian subjects over 65 years old with age-related sarcopenia, whereas Luo et al. [15] extended the investigation to adult patients aged 18 years and older, also including cases of secondary sarcopenia due to comorbidities and malignancies. From this perspective, although Onishi et al. [16] achieved an accuracy of 0.88 by applying CNNs to 3096 Japanese patients undergoing CT scans and Porciello et al. [32] identified three sarcopenia risk clusters in 879 cancer patients using PCA and k-means clustering, the transferability of these results to different contexts remains to be demonstrated.

Another critical issue concerns scalability: the acquisition of large amounts of data entails high costs and requires dedicated computational infrastructures, which are often unavailable in healthcare settings with limited resources. To overcome these obstacles, two strategies emerge with particular strength. The first consists of combining linear and non-linear algorithms, a well-established practice in data science that improves the robustness of predictions when dealing with complex and high-dimensional datasets.

The second strategy concerns the automation of data standardisation and annotation processes, thanks to structured frameworks and the use of Large Language Models (LLMs). LLMs are a promising example of this capability, as demonstrated by recent studies [77], which highlight their effectiveness in managing complex, large-scale databases, whose accurate annotation is crucial for training robust and reliable models [11]. In addition, LLMs facilitate the exploratory analysis of heterogeneous data, thus further advancing biomedical innovation.

It is worth noting that, in order to translate the potential of AI into diagnostic and therapeutic tools of real clinical value, it is necessary to adopt an integrated approach that combines methodological innovation, rigorous data standardisation and pragmatic implementation, thereby ensuring better outcomes for patients affected by sarcopenia. Notably, the explainability of the models via post hoc analysis, for example, by the SHAP algorithm, was used to enhance model interpretability. For instance, in [32], the authors highlighted serum albumin, nutritional status and diabetes as the key factors in predicting sarcopenia risk, thus aligning overall with the obtained outcomes. These findings underscore the importance of comprehensive patient profiling in improving sarcopenia assessment.

**Figure 1:**
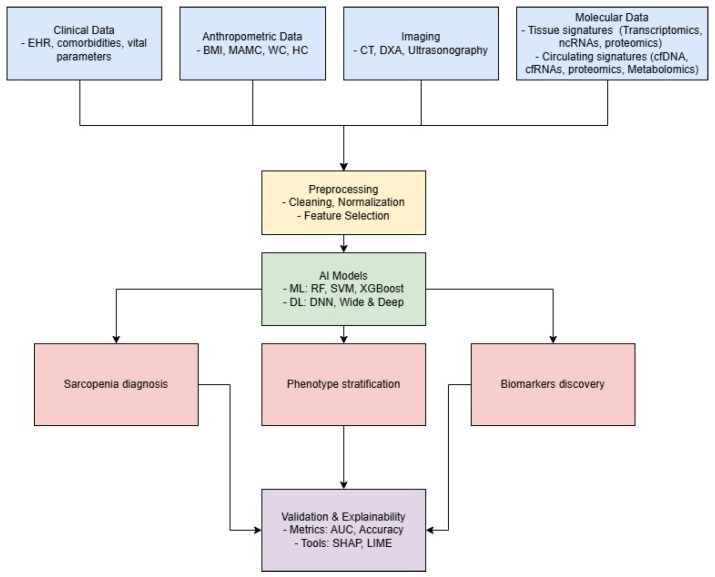
Proposed workflow for improving sarcopenia assessment by exploiting AI approaches.

Figure 1 illustrates our proposed workflow. It starts from the use of clinical, anthropometric, imaging and molecular data, which—once cleaned, normalised and subjected to feature selection—are processed by machine learning and deep learning models for diagnosis, phenotype stratification and the discovery of novel biomarkers, with validation via performance metrics and explainability tools.

## 7. Conclusions

AI can foster and speed up the identification of meaningful signatures of sarcopenia, from clinical data to molecular markers, as well as their integration into powerful, comprehensive models. Furthermore, AI can support data annotation and standardisation. However, although AI holds immense potential for improving sarcopenia diagnosis and management, most applications remain in developmental stages. Large-scale, multicentric clinical trials are crucial to establish the safety, efficacy and real-world applicability of these technologies. Continued interdisciplinary collaboration between clinicians, researchers, data scientists and policymakers will be key to unlocking AI’s full potential while safeguarding ethical and clinical standards, especially for the care of fragile patients.

## Figures and Tables

**Table 1:** Applications of AI for sarcopenia diagnosis.

Dataset	AI Model	Performance	Key Predictors	Ref.
EHR from 1304 patients	RF, SVM	AUC > 90%	Diagnoses, medications, lab tests	[15]
166 patients, 99 variables	RBF SVM	Accuracy: 82.5%, F1: 90.2%, precision: 82.8%	Age, hypertension, MNA, sodium	[18]
133 subjects, BPW signals	LDA, Scoring System	AUC: 0.77 (LDA), 0.83 (scoring)	APS	[17]
CHARLS	XGBoost	AUROC: 0.759	MMSE, drinking habits, BUN	[20]
WCHAT cohort, XMAT validation	Wide and Deep	AUC: 0.97, ACC: 91.1%	MAMC, CC, TSF, AST/ALT ratio	[29]
Italian ageing populations	RF (3 models)	ACC 89.89%, sensitivity 14.50%, specificity 99.37%	Albumin, CRP, vitamin D, folates	[22]
1510 patients	LR	S: 0.33, SO: 0.19 OS: 0.267	BMI	[24]
KNHANES (4020 patients)	LR, RF, SVM, GBM	AUC [men–women] RF: 0.82–0.78 SVM: 0.8–0.81 GB: 0.81–0.81 LR: 0.82–0.80	BMI, RBC, nutrient intake, water intake	[25]
3096 Japanese patients	CNN	ACC: 0.88	MAMC, CC, TSF, AST/ALT ratio	[16]
879 oncological patients	PCA + K-means	ACC: PC1 (59%), PC2 (24%), PC3 (15%)	Advanced age, lung, gynecological, gastroint. cancer, diabetes, malnutrition	[32]
231 post-surgical patients	XGBoost vs. LASSO	AUROC: 0.98	Serum albumin, diabetes, type surgery, nutritional score, ECOG status	[33]

**Table 2:** Molecular biomarkers and AI applications in sarcopenia.

Dataset	AI Model	Key Biomarkers	Performance	Ref.
-GSE1428 -GSE136344	LASSO, SVM-RFE	*MYH8*, *HOXB2*, *CDKN1A*	AUC > 0.7	[34]
-GSE111017	DNN (DSnet-v1)	27 AI-featured genes (e.g., *H4C3*, *PSMA6*, *CENPC*, *VPS35L*)	Acc. 0.96 Sens. 1.000 Spec. 0.94 AUC: 0.99	[14]
-GSE8479 -GSE9103 -GSE38718 -GSE1428	RF, ANN	*MT1X*, *CISD1*, *WISP2*	AUC: 0.999 (train), 0.85 (test)	[35]
509 Korean males	RFECV, Ensemble ML	8 CpG sites	AUC: 0.94	[38]

## Data Availability

Not applicable.
